# Public management approaches to an aging workforce: organizational strategies for strategies for adaptability and efficiency

**DOI:** 10.3389/fpsyg.2024.1439271

**Published:** 2024-07-25

**Authors:** Zouhengrui Wang, Jishan Fu, Weijun Bai

**Affiliations:** School of Public Management, Inner Mongolia University, Inner Mongolia Autonomous Region, Hohhot, China

**Keywords:** Chinese aged workers, psychological age climate, job motivation, organizational culture, autonomy in the workplace, social support

## Abstract

This study investigates the impact of psychological age climate on the motivation of aged workers in China and explores the mediating mechanisms at play. Two proposed chains of mediation capture the potential mechanisms underlying this process. The first chain involves the task and knowledge characteristics of work design, specifically autonomy arrangements and skill-based job demands, as mediators. The second chain focuses on the social and physical/contextual aspects of work design, including social support and ergonomic working conditions. The study sample consisted of 1,094 Chinese employees aged between 50 and 70 years (M = 55.66, SD = 4.274). Our findings reveal that a positive psychological age climate—organizational norms and practices that value and support older workers—significantly boosts their motivation to continue working. This enhancement in motivation is mediated by increased job autonomy and robust social support within the workplace, confirming that these elements are crucial for translating a positive age climate into tangible outcomes. Contrary to existing literature, our research does not support the mediating role of job design tailored to aged workers’ skills and ergonomic working conditions. This indicates that in the cultural and organizational context of China, where collective values and respect for elder wisdom predominate, autonomy and social support directly influence workers’ motivation more profoundly than ergonomic and job design considerations. The study underscores the importance of creating inclusive organizational cultures and implementing targeted support strategies to retain and engage aged workers effectively. It suggests that public policymakers and organizational leaders should focus on fostering positive psychological age climates and providing necessary autonomy and social resources to meet the unique needs of an aging workforce, thereby enhancing both individual and organizational outcomes in a globally aging society.

## Introduction

The global phenomenon of population aging poses significant challenges and opportunities for organizations across sectors, including the public service (Hedge and Borman, 2018; Bonzini et al., 2023). As lifespans increase and birth rates decline, the workforce is becoming increasingly older, with a growing proportion of employees nearing or exceeding traditional retirement ages (Ortar et al., 2023). This demographic shift is particularly pronounced in China, where the population is rapidly aging due to the combined effects of the one-child policy and increased life expectancy (Chen et al., 2023).

For public sector organizations, which are responsible for delivering essential services to citizens, effectively managing this aging workforce is of paramount importance. Extended working lives require public managers to employ older individuals successfully, that is, to keep them healthy, motivated, and productive (Van Woerkom et al., 2023). However, research suggests that many older employees in China perceive a biased organizational environment, where they are viewed as less competent, motivated, and productive compared to their younger counterparts (Yeung et al., 2021).

These negative age stereotypes can have a detrimental impact on the motivation of older workers to remain employed, potentially exacerbating labor shortages and skill gaps in the public sector (Truxillo et al., 2012; Zacher and Gielnik, 2014). Moreover, the traditional Chinese cultural emphasis on seniority and respect for elders may clash with the need to adapt to new ways of working, posing unique challenges for public managers (Zhang et al., 2023).

To address these challenges, researchers have identified a variety of organizational practices and strategies that may enable the successful employment of an aging and increasingly age-diverse workforce (Kulik et al., 2014). These include human resource policies, such as flexible work arrangements and training opportunities, as well as organizational cultures that value and support older workers.

Despite the extensive research on workforce aging, there is a noticeable gap in the literature concerning how public managers in China specifically can adapt to these demographic shifts (Komal et al., 2023). Existing studies often focus on Western contexts (Rudolph and Zacher, 2024) or private sector organizations (Li et al., 2021), leaving a critical need to explore the unique cultural and organizational dynamics at play in the Chinese public sector. This study seeks to fill this gap by investigating the role of psychological age climate, work design, and other organizational factors in shaping the motivation of older workers in the Chinese public sector.

The current study aims to explore how public managers in China can effectively adapt to demographic shifts, manage a diverse age range of employees, and maintain a high level of motivation to continue working beyond the traditional retirement age, while navigating the complexities of an aging workforce (Wang and Fang, 2020). By examining these factors, this research seeks to contribute new knowledge to the well-established field of public management and provide practical insights for policymakers and public sector leaders in China. Specifically, it will offer a nuanced understanding of how psychological age climate and work design influence older workers’ motivation, thus addressing the unique challenges posed by an aging workforce in the context of Chinese public service.

## Theoretical framework

### Workforce aging in Chinese public organizations

Population aging, defined as the increasing median age in the population due to declining birth rates and rising life expectancy, presents significant challenges and opportunities for organizations across sectors. This demographic shift is especially pronounced in China, as well as in other countries (Xiang et al., 2023).

The aging workforce phenomenon in China is influenced by several interrelated social, cultural, political, economic, and belief systems. Socially, the traditional Chinese value of filial piety places a strong emphasis on family responsibility toward elderly members, creating a unique dynamic in how older workers are perceived and supported within organizations (Liu et al., 2022). Culturally, the respect for seniority and age, deeply ingrained in Confucian principles, often clashes with the need for innovation and adaptability in modern work environments (Bai and Lei, 2020). Politically, the government’s policies, such as the one-child policy, have contributed to a shrinking younger workforce and an increasing dependency ratio, prompting the need for policies that encourage older individuals to remain in the workforce (Wei et al., 2020).

Economically, the rapid aging population poses challenges for sustaining economic growth, as a smaller working-age population may lead to labor shortages and decreased productivity (Park and Shin, 2023). The belief systems and indigenous values prevalent in Chinese society also play a critical role. The belief in the collective good and community well-being often drives organizational practices that support older workers, yet may also create resistance to change and adaptation of new management practices (Liu et al., 2022; Tian, 2023). Understanding these multifaceted influences is essential for public managers in China to develop strategies that effectively harness the potential of an aging workforce while mitigating the associated challenges.

The role of public management in addressing the challenges and harnessing the opportunities presented by an aging workforce seems crucial. Public sector organizations, which often provide essential services to citizens, must find ways to effectively manage this demographic transition. Extended working lives require organizations to employ older individuals successfully, that is, to keep them healthy, motivated, and productive (Ortar et al., 2023).

Researchers have named a variety of different organizational practices that may drive successful employment of an older and increasingly age-diverse workforce. These include HR policies such as flexible work arrangements, training and development opportunities, and phased retirement options (Uansin, 2023). Effective leadership and organizational culture that values and supports older workers also appear to be critical factors (Wilckens et al., 2023).

These endeavors seem particularly relevant in the context of Chinese public organizations, which face unique challenges in managing an aging workforce. The traditional Chinese cultural emphasis on seniority and respect for elders may clash with the need to adapt to new ways of working (Liou, 2024). Moreover, the highly bureaucratic nature of many Chinese public sector entities can hinder the implementation of age-inclusive policies and practices (Yao et al., 2024).

The current study is aimed at exploring how public managers in China can effectively adapt to demographic shifts, manage a diverse age range of employees, and maintain a high level of motivation to continue working, beyond the traditional retirement age, while navigating the complexities of an aging workforce. By examining the organizational factors and managerial strategies that enable successful aging in public sector settings, this research seeks to contribute to the growing body of literature on workforce aging and provide practical insights for policymakers and public sector leaders.

### Psychological age climate as predictor of motivation to continue working

A key factor that may influence the motivation of older workers to continue their employment is the psychological age climate (PAC) within the organization. PAC refers to the individual worker’s perception of how positively or negatively older workers are regarded in their work environment (Kilroy et al., 2022). This construct is distinct from organizational age climate, which captures the shared perceptions of age-related attitudes across the entire organization (Anderson and West, 1998).

Research has shown that many older employees perceive a biased organizational environment, where they are viewed as less competent, motivated, and productive compared to their younger counterparts (Posthuma and Campion, 2009; Finkelstein et al., 2015). This negative perception of one’s work environment can have a detrimental effect on the motivation of older workers to remain employed (Axelrad et al., 2023; Bellotti et al., 2023).

In line with Rhodes (1983) assessment that the work environment, including factors such as co-worker relations and age-related expectations, influences the work attitudes and behaviors of older workers, past studies have revealed that PAC is meaningfully related to work-related outcomes. Specifically, research has shown that the more positively older workers perceive the age climate in their organization, the more committed they are to their employer and the more motivated they are to continue working (Rabl, 2010).

This relationship can be explained by social identity theory, which posits that individuals strive to maintain a positive self-image and will seek out groups and environments that reinforce their self-worth (Brown, 2020). When older workers perceive their organization as valuing and respecting older employees, it fulfills their need for a positive social identity, which in turn enhances their motivation to remain engaged and productive in their roles.

Conversely, a negative PAC, characterized by the endorsement of age stereotypes and a lack of age-inclusive policies and practices, can lead older workers to feel devalued and marginalized within the organization. This can undermine their motivation to continue working, as they may perceive limited opportunities for growth and development (Truxillo et al., 2012; Zacher and Gielnik, 2014).

Most recent studies have continued exploration into the psychological age climate and motivation among aged workers, revealing several significant insights. Froehlich et al. (2023) emphasizes that a proactive personality can greatly enhance the motivation and intention to learn in older employees. Conversely, negative age stereotypes, when institutionalized, can severely diminish their motivation. This underscores the importance of fostering supportive environments that promote continuous learning and development for older workers.

In a systematic review, Bratun et al. (2023) found that workers who choose to continue their employment beyond the typical retirement age are driven by motives related to doing, being, becoming, and belonging. Health emerged as a critical factor influencing their decision to remain in the workforce, suggesting that maintaining good health can be a strong motivator for continued employment among aged workers.

In the Chinese context, Xu et al. (2022) reveals that psychological safety and intrinsic motivation are crucial mediators between organizational climate, innovation orientation, and innovative work behavior. Although this study focused on high-tech SMEs, the findings are broadly applicable, highlighting the importance of creating a supportive work environment for aged workers to foster innovation and engagement.

Furthermore, research by Oshio (2021) indicates that job dissatisfaction among middle-aged workers is linked to higher risks of psychological distress, poor self-rated health, and health-related resignation. This finding underscores the need for monitoring and addressing job dissatisfaction to promote occupational health and well-being among aged workers.

Collectively, these studies suggest that psychological factors, organizational climate, and health considerations play pivotal roles in the motivation and well-being of aged workers. Creating a positive age climate and addressing health and satisfaction issues are essential for maintaining an engaged and productive older workforce.

Given the importance of sustaining an engaged and productive older workforce, particularly in the public sector where the population is aging rapidly, understanding the role of PAC in shaping the motivation of older employees to remain in the workforce is a crucial area of inquiry. The current study aims to further explore this relationship within the context of Chinese public organizations, where cultural norms and organizational structures may present unique challenges in cultivating an age-friendly work environment.

### Aged workers-friendly work design: mediating role between psychological age climate and motivation to continue working

The relationship between psychological age climate and the motivation of older workers to continue working may be mediated by the design of work to better accommodate the needs and capabilities of aging employees. Work design, which describes how jobs, tasks, and roles are structured and enacted, has been recognized as a critical factor in retaining and engaging older workers (Morgeson and Humphrey, 2006).

Specifically, research has identified several dimensions of work design that are particularly relevant for addressing the challenges of an aging workforce (Hertel et al., 2013; Finkelstein et al., 2021). These include, first, the task characteristics: Increasing autonomy and control over work tasks can empower older workers and allow them to leverage their experience and expertise (Finsel et al., 2023). Second, the Knowledge characteristics: Adapting job demands to match the skills and abilities of older workers, such as through training and development opportunities, can enhance their motivation and productivity (Maurer et al., 2003). Third, the Social characteristics: Providing strong social support and mentoring from co-workers and supervisors can help older employees feel valued and engaged (Bal et al., 2008; Kooij et al., 2014). And, finally, the Physical/contextual characteristics: Ensuring ergonomic working conditions that accommodate the physical needs of aging workers can improve their comfort and well-being (Cadiz et al., 2019).

These work design practices that are tailored to the needs of older employees, which we refer to as “aged workers-friendly work design,” may serve as a critical mediating mechanism between psychological age climate and the motivation of older workers to continue working. When older workers perceive a positive age climate in their organization, where they are respected and valued for their contributions, they are more likely to experience a work environment that is designed to support their unique needs and capabilities. This, in turn, can enhance their motivation to remain engaged and productive, as they feel empowered, challenged, and supported in their roles (Chen et al., 2023).

Conversely, a negative psychological age climate, characterized by age discrimination and the endorsement of negative stereotypes, may lead to work designs that do not adequately address the requirements of older workers, undermining their motivation to continue working (Cox and Young, 2020).

Two proposed chains of mediation capture the potential mechanisms underlying this process. The first chain involves the task and knowledge characteristics of work design, specifically autonomy arrangements and skill-based job demands, as mediators. The second chain focuses on the social and physical/contextual aspects of work design, including social support and ergonomic working conditions. Given that the two first categories (task and knowledge) are strongly related, and the other two are more contextual oriented (one to the social context and the other to the physical context of work), two chain mediations are proposed: M1 (Autonomy arrangements for aged workers) and M2 (Work design according to aged workers’ Skills), on the one hand, and M3 (social support for aged workers), and M4 (aged workers-oriented ergonomic conditions).

**Figure 1:**
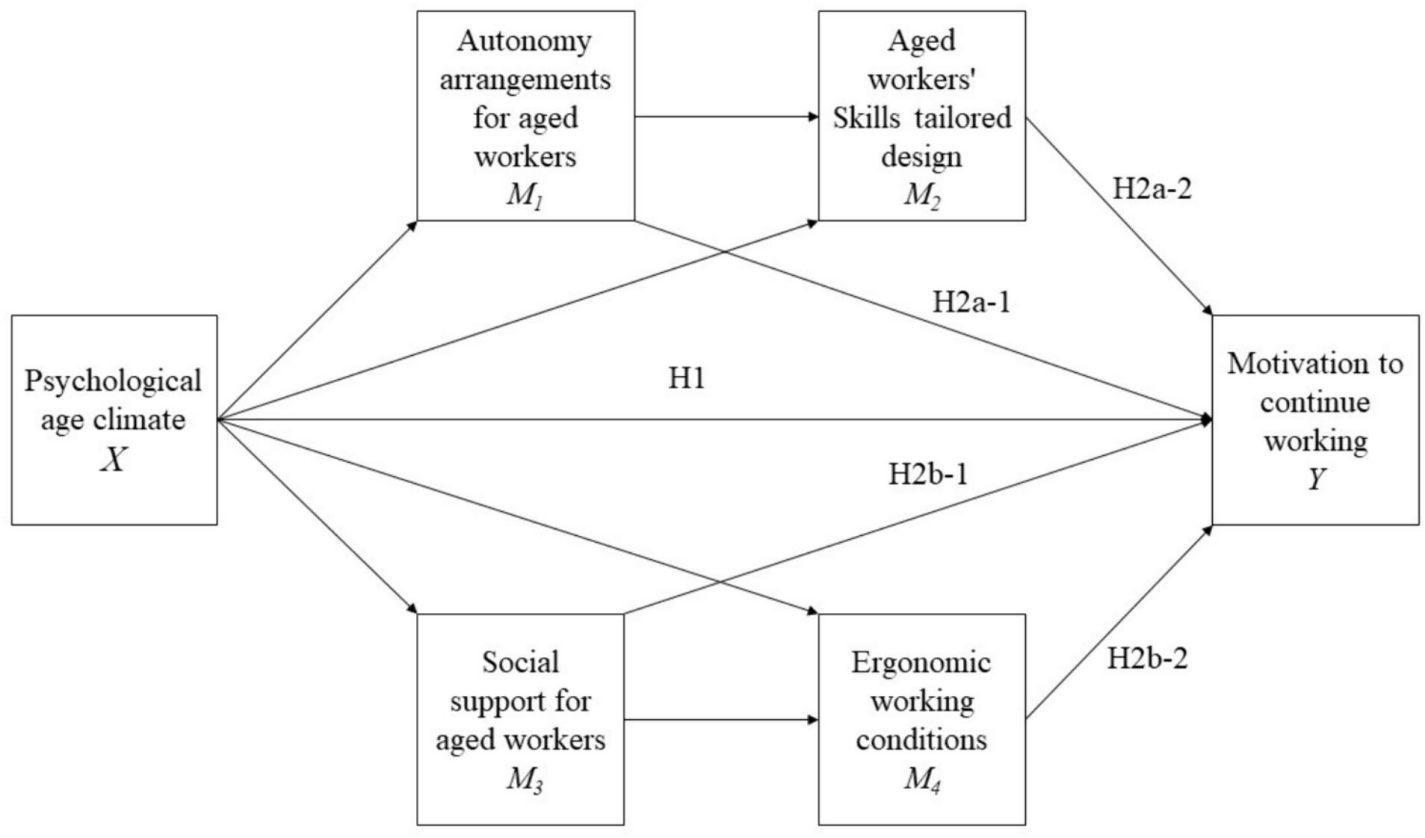
Hypotheses and theoretical model.

By examining these mediating pathways, the current study aims to provide a more nuanced understanding of how organizational age climate shapes the motivation of older workers in Chinese public sector organizations, through the design of work that leverages the strengths and addresses the needs of an aging workforce (Figure 1).

## Hypotheses

Based on a thorough review of previous literature, we developed the following hypotheses:

*H1*: Psychological age climate (PAC) has a direct positive effect on the motivation to continue working among older workers. Previous studies have shown that an inclusive and supportive psychological age climate can enhance the motivation of older employees (Zacher and Gielnik, 2014; Xu et al., 2022; Bratun et al., 2023).

*H2a*: The relationship between PAC and motivation to continue working is mediated by aged workers-friendly task and knowledge characteristics of work design. Research indicates that offering flexible work options and increasing knowledge characteristics of work design helps older workers maintain productivity by accommodating their needs and preferences (Hertel et al., 2013; Finkelstein et al., 2021). Hence, the following hypotheses are proposed:

*H2a-1*: Autonomy arrangements for aged workers mediates the relationship between PAC and motivation to continue working.

*H2a-2*: Aged workers’ skills tailored design mediates the relationship between PAC and motivation to continue working.

*H2b*: The relationship between PAC and motivation to continue working is mediated by aged workers-friendly social and physical/contextual characteristics of work design. Prior research suggests that supportive work designs, which include both social aspects (such as collegial support) and physical/contextual aspects (such as ergonomic adjustments), enhance the motivation of older employees to remain in the workforce (Bal et al., 2008; Kulik et al., 2014; Cadiz et al., 2019). Hence, specifically, we proposed that:

*H2b-1*: Social support for aged workers mediates the relationship between PAC and motivation to continue working.

*H2b-2*: Ergonomic working conditions tailored to aged workers mediates the relationship between PAC and motivation to continue working.

**Figure 2:**
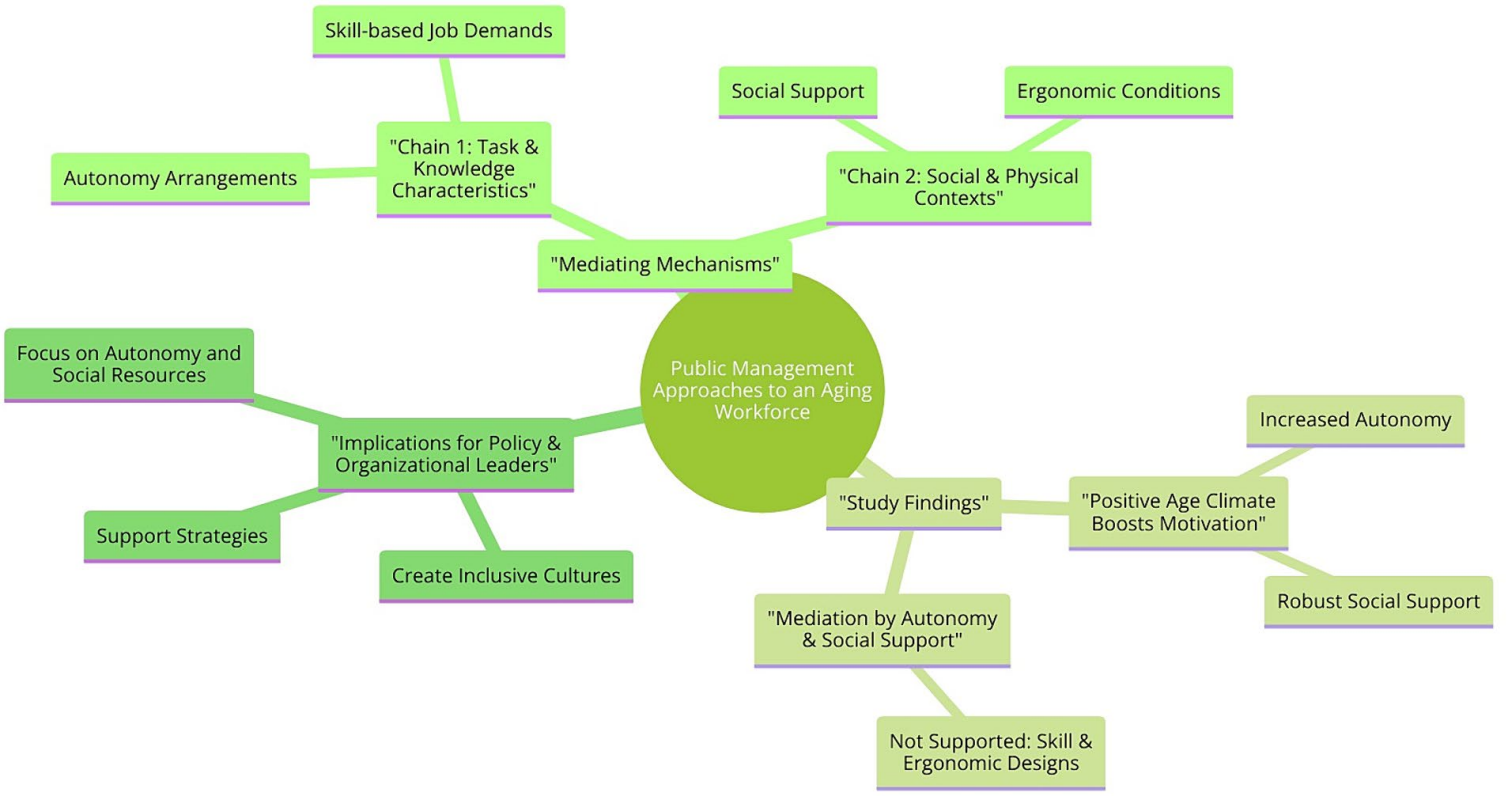
Research model.

These hypotheses capture the proposed direct and indirect relationships between psychological age climate, aged workers-friendly work design, and the motivation of older employees to continue working, as depicted in the research model (Figure 2).

## Method

### Participants

The research sample comprised 1,094 Chinese employees aged between 50 and 70 years, with a mean age of 55.66 years (SD = 4.274). The gender distribution was nearly equal, with 49.2% (*n* = 538) being men and 50.8% (*n* = 556) being women. Participants were selected from various professional categories: management (10.2%), middle-level management (28.5%), technician and administrative clerk positions (46.9%), and non-qualified roles (14.4%).

The study primarily focused on individuals employed in the public sector, encompassing diverse sectors such as education (15.8%), healthcare (14.4%), and less represented fields including state security, social insurance, and public services. Educational backgrounds varied significantly among participants, with 45.5% holding a university degree, 23.5% possessing a high school diploma, 15.9% having vocational training, 14.5% completing compulsory schooling, and a small fraction (0.5%) attaining a PhD.

Seniority within their respective companies ranged from new employees to those with up to 45 years of service, averaging around 21.96 years (SD = 11.002). This data provided valuable insights into the relationship between job tenure, satisfaction, and career advancement in the public sector.

### Instruments

#### Psychological age climate

Participants’ perceptions of the psychological age climate (PAC) in their organization were assessed using the 12-item Psychological Age Climate Scale (PACS) developed by Noack (2009). The PACS measures the degree to which older workers are viewed positively within the organization, capturing three broad types of age stereotypes: competence, motivation, and productivity. Participants indicated the extent to which they agreed or disagreed with each adjective (e.g., “competent,” “motivated,” “productive”) on a 5-point Likert scale ranging from 1 (Disagree) to 5 (Agree). All items were positively worded to minimize social desirability bias. Higher scores on the PACS indicate a more positive psychological age climate.

#### Aged workers-friendly work design

The design of participants’ work was measured using a multidimensional Work Design Questionnaire (Morgeson and Humphrey, 2006). The WDQ assesses four key aspects of work design: Task characteristics: Capturing the nature and variety of tasks, as well as the level of autonomy and control over work. Knowledge characteristics: Reflecting the cognitive demands and skill requirements of the job. Social characteristics: Addressing the social environment and interpersonal aspects of the work. Physical/contextual characteristics: Pertaining to the physical and environmental conditions in which the work is performed. Participants rated the extent to which each of these work design dimensions was present in their job on a 5-point Likert scale ranging from 1 (Strongly disagree) to 5 (Strongly agree), with higher scores indicating a more favorable work design.

#### Motivation to continue working

Participants’ motivation to continue working beyond the official retirement age was measured using a 3-item scale adapted from Armstrong-Stassen (2008). Example items include “If I were completely free to choose, I would prefer to continue working after my official retirement age” and “I expect to continue working as long as possible after my official retirement age.” Responses were provided on a 5-point Likert scale from 1 (Strongly disagree) to 5 (Strongly agree), with higher scores reflecting greater motivation to continue working. The full list of items of each scale is included in the Table A1.

### Procedure

The Ethical Committee of Inner Mongolia University approved the study. A comprehensive survey was designed to gather demographic information, professional details, and job satisfaction levels among employees aged 50 to 70 in the public sector. The survey was administered online to ensure voluntary participation and confidentiality, enhancing accessibility and response rates given the age range of the participants. Participants were selected based on the following inclusion criteria: they had to be Chinese employees aged between 50 and 70 years, currently employed in the public sector, including fields such as education, healthcare, state security, social insurance, and public services. They could hold various job roles, including management, middle-level management, technician, administrative clerk positions, and non-qualified personnel. Educational backgrounds were diverse, ranging from compulsory schooling to PhDs. The exclusion criteria for the study are as follows: individuals who are below 50 or above 70 years old, and those who are not currently employed, are excluded from participation.

The data collection was performed over a three-month period, allowing ample time for participants to respond. An informed consent process was initiated at the start of the survey, ensuring that participants were aware of the study’s scope and their rights. Data analysis followed the completion of the data collection phase, focusing on identifying trends and patterns relevant to the research hypotheses concerning age, professional experience, and sector-specific challenges and opportunities.

### Data analyses

Descriptive and correlational analyses were conducted using SPSS version 24. For the mediation analyses, we employed the Process macro for SPSS (version 4.2). The mediation analysis utilized Model 82 of the Process macro (Hayes, 2013, 2022). This model tested a chain mediation in which the mediating role of age-friendly work design was examined in the relationship between Psychological Age Climate and Motivation to Continue Working. Specifically, the mediation was explored through two pathways: the first pathway involved autonomy arrangements and skills adaptation, while the second pathway included social support and ergonomic working conditions tailored to aged workers. 275-277.Before conducting the mediation analysis, we tested for several assumptions, including the normality of the distribution of variables, linearity, homoscedasticity, and the absence of multicollinearity among the predictors. The analysis adhered to the assumption that zero must be excluded from the 95% bias-corrected confidence interval to support the chain mediation hypothesis. If this condition is met, it indicates that the parameter significantly differs from zero at *p* < 0.05. The mediation analysis was further validated using 5,000 bootstrap re-samples, which provided estimates of the indirect effects.

## Results

### Descriptive statistics and Pearson’s correlations

The analysis of the data yielded several noteworthy relationships among the variables studied. The Psychological Age Climate within the organization was observed to have a considerable association with the intention of aged workers to continue their employment. This suggests that the way aging is perceived and managed within the workplace context has a significant impact on workers’ decisions to remain in the labor force. Additionally, the autonomy afforded to aged workers was correlated with their intention to continue working, implying that the degree of control and independence experienced by these workers may influence their commitment and motivation to remain employed. Skill adaptation for aged workers also showed a relationship with the intention to continue working, although to a lesser extent compared to autonomy. This points to the relevance of matching workers’ skills with job demands, particularly for the aging workforce. Social support for aged workers was similarly related to their continued working intention. Moreover, ergonomic working conditions tailored to aged workers were linked to their motivation to continue working.

In examining the interrelationships among the variables, it was evident that autonomy, skill adaptation, and social support are interrelated factors, each significantly associated with the others (Table 1).

**Table 1:** Descriptive statistics and correlation matrix for key study variables (*N* = 1,094).

Variable	Mean	SD	1	2	3	4	5	6
1. Psychological age climate	3.53	1.01	*0.72*	-	-	-	-	-
2. Autonomy	3.92	1.68	0.291**	*0.78*	-	-	-	-
3. Skill adaptation	3.42	1.62	0.161**	0.558**	*0.79*	-	-	-
4. Social support	3.27	1.46	0.290**	0.663**	0.635**	*0.84*	-	-
5. Ergonomic conditions	4.44	1.80	0.214**	0.527**	0.450**	0.450**	*0.70*	-
6. Continued working intention	3.65	0.99	0.698**	0.434**	0.242**	0.381**	0.279**	*0.69*

**p < 0.01. Correlations are Pearson’s r. Values in italics in the diagonal are Cronbach’s Alphas.

### Hypothesis testing

The results from the total effect model showed that Psychological age climate had a significant positive direct effect on the motivation to continue working (*b* = 0.6832, *p* < 0.001). Therefore, H1 was supported (Table 2).

**Table 2:** Regression analysis for motivation to continue working predicted by psychological age climate and other mediators.

Predictor	*B*	SE	*t*	*p*	95% CI (LLCI, ULCI)	*β*
Constant	0.889	0.084	10.64	< 0.001	[0.725, 1.053]	
Psychological age climate	0.603	0.021	28.51	<0.001	[0.562, 0.645]	0.616
Autonomy	0.128	0.018	7.35	<0.001	[0.094, 0.163]	0.218
Skill adaptation	−0.018	0.017	−1.07	0.284	[−0.051, 0.015]	−0.030
Social support	0.047	0.021	2.29	0.022	[0.007, 0.088]	0.070
Ergonomic conditions	0.008	0.014	0.60	0.551	[−0.019, 0.035]	0.015

*R*^2^ = 0.5479; *F* (5, 1,088) = 263.68; *p* < 0.001.

*H2a*: The relationship between Psychological age climate and motivation to continue working is mediated by aged workers-friendly task and knowledge characteristics of work design.

*H2a-1*: Autonomy arrangements for aged workers mediates the relationship between Psychological age climate and motivation to continue working.

The results indicated that Psychological age climate had a significant positive effect on autonomy arrangements for aged workers (*b* = 0.4834, *p* < 0.001). Autonomy arrangements, in turn, had a significant positive effect on motivation to continue working (*b* = 0.1283, *p* < 0.001). The indirect effect of PAC on motivation through autonomy was also significant [indirect effect = 0.0620, 95% CI (0.0407, 0.0851)]. Thus, H2a-1 was supported (Table 3).

**Table 3:** Results for autonomy arrangements for aged workers predicted by psychological age climate.

Predictor	*B*	SE	*t*	*p*	95% CI (LLCI, ULCI)
Constant	2.218	0.177	12.56	0.000	[1.872, 2.565]
Psychological age climate	0.483	0.048	10.04	0.000	[0.389, 0.578]

*R*^2^ = 0.0846; *F* (1, 1,092) = 100.88; *p* < 0.001.

*H2a-2*: Work design adapted to aged workers’ skills mediates the relationship between PAC and motivation to continue working.

While Psychological age climate had a significant positive effect on work design adapted to aged workers’ skills (*b* = 0.5402, *p* < 0.001), this work design characteristic did not have a significant effect on motivation to continue working (*b* = −0.0180, *p* = 0.284). The indirect effect of Psychological age climate on motivation through work design adapted to aged workers’ skills was not significant (indirect effect = 0.0000, 95% CI [−0.0021, 0.0024]). Therefore, H2a-2 was not supported (Table 4).

**Table 4:** Results for analysis for skills tailored design for aged workers predicted by psychological age climate and autonomy arrangements.

Predictor	B	SE	*t*	*p*	95% CI (LLCI, ULCI)
Constant	1.303	0.159	8.22	0.000	[0.992, 1.615]
Psychological Age Climate	−0.002	0.042	−0.04	0.965	[−0.085, 0.081]
Autonomy	0.540	0.025	21.27	0.000	[0.490, 0.590]

*R*^2^ = 0.3116; *F* (2, 1,091) = 246.90; *p* < 0.001.

*H2b*: The relationship between Psychological age climate and motivation to continue working is mediated by aged workers-friendly social and physical/contextual characteristics of work design (Tables 5, 6).

**Table 5:** Results for social support for aged workers predicted by psychological age climate.

Predictor	*B*	SE	*t*	*p*	95% CI (LLCI, ULCI)
Constant	1.791	0.154	11.65	0.000	[1.489, 2.093]
Psychological age climate	0.420	0.042	10.03	0.000	[0.338, 0.502]

*R*^2^ = 0.0843; *F* (1, 1,092) = 100.57; *p* < 0.001.

**Table 6:** Results for ergonomic working conditions predicted by social support and psychological age climate.

Predictor	*B*	SE	*t*	*p*	95% CI (LLCI, ULCI)
Constant	2.168	0.186	11.65	0.000	[1.803, 2.534]
Psychological age climate	0.161	0.050	3.22	0.001	[0.063, 0.259]
Social support	0.521	0.035	15.08	0.000	[0.453, 0.589]

*R*^2^ = 0.2104; *F* (2, 1,091) = 145.31; *p* < 0.001.

*H2b-1*: Social support for aged workers mediates the relationship between Psychological age climate and motivation to continue working.

The results showed that Psychological age climate had a significant positive effect on social support for aged workers (*b* = 0.4202, *p* < 0.001). Social support, in turn, had a significant positive effect on motivation to continue working (*b* = 0.0472, *p* = 0.022). The indirect effect of PAC on motivation through social support was also significant [indirect effect = 0.0198, 95% CI (0.0031, 0.0387)]. Thus, H2b-1 was supported.

*H2b-2*: Ergonomic working conditions tailored to aged workers mediates the relationship between Psychological age climate and motivation to continue working.

The results showed that Psychological age climate had a significant positive effect on ergonomic working conditions tailored to aged workers (*b* = 0.1610, *p* = 0.001). Ergonomic working conditions, however, did not have a significant effect on motivation to continue working (*b* = 0.0081, *p* = 0.551). The indirect effect of Psychological age climate on motivation through ergonomic working conditions was not significant [indirect effect = 0.0013, 95% CI (−0.0037, 0.0069)]. Therefore, H2b-2 was not supported (Table 7).

**Table 7:** Indirect effects of psychological age climate on motivation to continue working.

Path	Effect	BootSE	BootLLCI	BootULCI	c’cs
TOTAL	0.0803	0.0107	0.0599	0.1026	0.0820
Psychological Age Climate → Autonomy → Motivation Continue Working	0.0620	0.0111	0.0407	0.0851	0.0633
Psychological Age Climate→ Skill Adaptation→ Motivation Continue Working	0.0000	0.0010	−0.0021	0.0024	0.0000
Psychological Age Climate→ Social Support→ Motivation Continue Working	0.0198	0.0090	0.0031	0.0387	0.0203
Psychological Age Climate→ Ergonomic conditions → Motivation Continue Working	0.0013	0.0026	−0.0037	0.0069	0.0013
Psychological Age Climate→ Autonomy → Skill Adaptation→ Motivation Continue Working	−0.0047	0.0040	−0.0126	0.0033	−0.0048
Psychological Age Climate→ Social Support→ Ergonomic conditions → Motivation Continue Working	0.0018	0.0033	−0.0047	0.0083	0.0018

Bootstrapping with 5,000 samples was used to estimate the indirect effects, providing a robust assessment of these pathways.

In summary, the findings partially supported the proposed mediation hypotheses. Autonomy arrangements for aged workers and social support for aged workers were found to mediate the relationship between Psychological age climate and motivation to continue working, while work design adapted to aged workers’ skills and ergonomic working conditions did not serve as significant mediators.

## Discussion

The current study examined the influence of psychological age climate on the motivation of Chinese aged workers to continue working, as well as the mediating mechanisms through which this relationship operates. The findings provide important insights for public management and organizational approaches to supporting an aging workforce.

The results demonstrate that a positive psychological age climate, which reflects organizational norms and practices that value and support older workers, has a significant direct effect on enhancing the motivation of aged workers to continue working. This supports the notion that cultivating an organizational culture that is inclusive and accommodating of older employees can be an effective strategy for retaining this segment of the workforce (Hertel and Zacher, 2018; Van der Heijden et al., 2020). As the global population continues to age, public policymakers and organizational leaders must prioritize fostering work environments that recognize and leverage the valuable skills and experience of older workers (Barakovic Husic et al., 2020).

The study also identified several chain mediating pathways through which psychological age climate influences motivation to continue working. Specifically, the findings indicate that autonomy arrangements for aged workers and social support for aged workers serve as important mechanisms linking psychological age climate to enhanced work motivation (Maalaoui et al., 2020). This suggests that providing aged workers with greater job autonomy and social resources within the workplace can help translate a positive age climate into tangible outcomes, such as a stronger desire to remain employed (Brochado, 2020).

The current study’s findings on the influence of psychological age climate on the motivation of Chinese aged workers to continue working align with several previous studies, while also providing some novel insights. In this vein, Hertel and Zacher (2018) examined the impact of age-inclusive HR practices on the retention of older workers across multiple countries. Their findings corroborate the present study’s emphasis on the importance of cultivating an organizational culture that values and supports older employees. They found that age-inclusive climates, characterized by positive attitudes toward older workers and flexible work arrangements, were positively associated with the motivation and retention of aged workers.

Similarly, Wilckens et al. (2021) suggested the relationship between age-friendly work environments and the job satisfaction and turnover intentions of older workers. Their results also highlighted the critical role of a positive psychological age climate in enhancing the work-related well-being and retention of aged employees. These studies, conducted in different cultural contexts, lend further support to the current study’s core findings (Cooke et al., 2020).

Interestingly, the current study did not find support for the mediating roles of work design adapted to aged workers’ skills and ergonomic working conditions (Jonsson et al., 2020). This suggests that while job design characteristics and physical work environments are important considerations for supporting an aging workforce, they may not be the primary pathways through which psychological age climate influences motivation to continue working. It is possible that the direct provision of autonomy and social support has a more proximal impact on aged workers’ motivation and engagement (Park and Lee, 2020).

The current study’s lack of support for the mediating roles of work design adapted to aged workers’ skills and ergonomic working conditions diverges from some previous research. For instance, a study by Shantz et al. (2016) found that job design characteristics, such as task variety and autonomy, were important mechanisms through which organizational support for older workers influenced their motivation and performance. Additionally, research by Andersen et al. (2021) has emphasized the importance of ergonomic work environments in promoting the well-being and productivity of older workers. One possible explanation for these differences may be the specific cultural and organizational contexts in which the studies were conducted (Nilsson, 2020).

A possible reason why work design adapted to aged workers’ skills and ergonomic working conditions were not significant mediators might be due to the complex interplay of various factors that influence motivation and well-being among older workers. While ergonomic adjustments and tailored work designs are crucial for physical comfort and reducing strain, they may not directly address psychological and emotional needs that significantly impact motivation. For instance, factors such as perceived respect, recognition, opportunities for personal growth, and social inclusion might play a more substantial role in mediating motivation and well-being. Additionally, older workers may prioritize intrinsic motivations, such as personal fulfillment and the desire to contribute meaningfully, over extrinsic factors like work design. Furthermore, the effectiveness of ergonomic interventions and skill adaptations can be limited if they are not accompanied by a supportive organizational culture and management practices that value and leverage the experience and knowledge of older employees. Hence, while ergonomics and skill adaptations are important, they may not be sufficient on their own to significantly mediate the broader aspects of aged workers’ motivation and well-being.

Cultural, organizational, and methodological factors may also explain why work design adapted to aged workers’ skills and ergonomic working conditions were not significant mediators. Culturally, there might be varying levels of value and respect for older workers across different societies, which can influence how they perceive and respond to workplace adaptations. In some cultures, older workers might face age-related stigma that undermines the positive impacts of ergonomic adjustments. Organizationally, the presence of a supportive culture that values inclusivity and continuous development may be more critical for motivation than physical adjustments alone. If an organization does not foster an environment that promotes respect, recognition, and social integration, even well-designed ergonomic interventions might fail to boost motivation. Methodologically, the way these factors are measured and analyzed can affect findings. For instance, self-reported data on job satisfaction and motivation can be influenced by subjective perceptions, which might not accurately capture the real impact of ergonomic improvements. Additionally, the duration of the study and the specific metrics used to assess work design and ergonomic conditions might not fully encapsulate their long-term effects on motivation and well-being. Thus, considering these cultural, organizational, and methodological dimensions is essential to understanding the complexities behind these findings.

The current study’s focus on the Chinese workforce may have revealed unique dynamics or priorities that were not as prominent in previous research. It is also possible that in the current study, the direct provision of autonomy and social support had a more immediate and salient impact on aged workers’ motivation, overshadowing the mediating effects of job design and physical work environments (Yan et al., 2011).

There are several potential cultural and organizational factors that may explain the differences in findings regarding the mediating roles of job design and physical work environments between the current study and previous research (Cooke et al., 2021). From a cultural perspective, the current study was conducted in China, a country characterized by a more collectivist cultural orientation, whereas much of the previous research has been carried out in more individualistic Western societies. In collectivist cultures, the emphasis on group harmony and interdependence may make the social support and autonomy aspects of the work environment particularly salient for older workers’ motivation (Hu et al., 2024).

Additionally, in some Asian cultures, including China, there is a stronger cultural emphasis on respecting and deferring to the wisdom and experience of older individuals. This cultural value may make the psychological age climate and social support mechanisms more influential than job design factors. Furthermore, older workers in different cultural contexts may have varying preferences and needs regarding work-life balance, and in China, the importance of family responsibilities and caregiving duties for older adults may overshadow the significance of physical work environments compared to Western contexts (Zhao et al., 2021).

Organizational factors may also play a role in the observed differences. The current study’s sample may have been drawn from industries or occupations where physical job design and ergonomics are less critical determinants of work motivation compared to the samples in previous studies. Additionally, older, more established organizations may have the resources and infrastructure to provide robust ergonomic support and job design accommodations for older workers, whereas younger or smaller organizations may prioritize more immediate social and autonomy-related factors (Tan et al., 2022).

Moreover, in rapidly evolving organizational contexts, the ability to maintain a positive psychological age climate and provide social support may be more crucial for retaining older workers than ensuring optimal job design or physical work environments, which can be more resource-intensive to implement. Finally, certain industries or sectors may have more well-established norms and practices around accommodating the needs of older workers, thus reducing the mediating effects of job design and physical work environments observed in the current study (Chen et al., 2024).

Understanding these cultural and organizational factors is crucial for public policymakers and organizational leaders to develop tailored, effective strategies for supporting and retaining their aging workforces. The current findings should be compared with recent studies from other countries (Cybulski et al., 2020) or with transnational results (Beard et al., 2016) that highlight that older individuals who remain motivated in their self-development, such as through studying, typically exhibit mild depressive and anxiety symptoms. The mental health of Polish participants in the Third Age University program was significantly influenced by their socio-occupational and marital status, as well as their financial condition. The study revealed that individuals with probable alcohol use problems differed from other respondents in terms of the prevalence of possible bipolar disorder, depression, non-psychotic symptoms of mental disorders, as well as their levels of state and trait anxiety and anger control. Despite these nuances, the overall body of research suggests that a multifaceted approach, addressing both organizational climate and job-related factors, is most effective in supporting and retaining an aging workforce. Overall, this study highlights the importance of public management and organizational strategies that address the unique needs and preferences of an aging workforce. By fostering positive psychological age climates and providing targeted support for autonomy and social connections, public and private sector leaders can more effectively retain and engage older workers, ultimately benefiting both individuals and organizations.

### Limitations of the present study and potential avenues for research

While the current study provides valuable insights into the influence of psychological age climate on the motivation of Chinese aged workers to continue working, it is important to acknowledge its limitations and identify potential avenues for future research.

One key limitation of the study is its cross-sectional design, which limits the ability to infer causal relationships between the variables. Future research could adopt a longitudinal approach to track changes in psychological age climate, work design factors, and motivation to continue working overtime, providing a more robust understanding of the dynamic nature of these relationships. This would also help disentangle the directionality of the observed effects and shed light on the potential for reciprocal relationships between the study variables.

The current study relied solely on self-reported data from aged workers, which introduces potential biases and common method variance. Self-reported data can be influenced by subjective perceptions, social desirability bias, and recall bias, potentially skewing the results. The cross-sectional nature of the study further limits the ability to draw causal inferences, as it captures data at a single point in time, making it challenging to establish the directionality of relationships.

To address these limitations, future research should incorporate longitudinal designs that track changes over time, providing a clearer picture of the causal relationships between psychological age climate and organizational outcomes. Longitudinal studies can help identify trends, long-term effects, and the stability of observed relationships. Additionally, adopting mixed-methods approaches that combine quantitative and qualitative data can offer a more nuanced understanding of the phenomena under study. For instance, integrating interviews or focus groups with survey data can provide deeper insights into the experiences and perspectives of older workers.

Incorporating objective measures alongside self-reported data can also strengthen the validity of findings. Including metrics such as work performance evaluations, absenteeism records, and actual retirement behavior can provide a more comprehensive understanding of the impact of a positive psychological age climate on organizational outcomes. By triangulating data from multiple sources, researchers can mitigate the biases inherent in self-reports and enhance the robustness of their conclusions. Through these methodological enhancements, future studies can provide more reliable and actionable insights into the dynamics of age-related factors in the workplace.

Furthermore, the study was conducted exclusively within the Chinese public sector context. While this provides valuable insights into the experiences of aged workers in a specific cultural and organizational setting, it also limits the generalizability of the findings. The unique characteristics of the Chinese public sector, including its cultural, institutional, and organizational dynamics, may not be directly applicable to other settings. To better understand the broader applicability of these findings, future research should consider comparative studies across different countries, industries, and organizational settings. Such studies could uncover the nuanced interplay of cultural, institutional, and organizational factors in shaping the motivation of older workers (Da Silva and Helal, 2022; St-Onge et al., 2022). Exploring these diverse contexts will help to determine whether the observed relationships hold true universally or are specific to particular environments, thereby enhancing the robustness and relevance of the research.

Another limitation is the lack of in-depth qualitative data that could provide a richer, contextualized understanding of the lived experiences and perspectives of aged workers. Future research could employ a mixed-methods approach, incorporating qualitative interviews or focus groups, to gain deeper insights into the mechanisms underlying the observed relationships and to uncover any additional factors that may influence the motivation of older workers.

Finally, the current study focused on the psychological age climate and work design factors as predictors of motivation to continue working. However, there may be other individual, interpersonal, and organizational factors that could influence this outcome, such as financial considerations, health status, family responsibilities, and organizational support for retirement planning. Incorporating a more comprehensive set of predictors could yield a more holistic understanding of the complex dynamics shaping the motivation of aged workers.

Despite these limitations, the current study contributes to the growing body of research on supporting and retaining an aging workforce. By highlighting the importance of a positive psychological age climate and the mediating roles of autonomy and social support, the findings provide valuable guidance for public policymakers and organizational leaders seeking to develop effective strategies for engaging and empowering their older employees.

To address the limitations of the current study and enhance the understanding of aged workers’ motivation, future research should be conducted in diverse cultural and organizational settings. Investigating aged workers’ experiences and motivations across various industries and countries can provide a more comprehensive and nuanced perspective. Cross-cultural comparisons can reveal how different societal values, economic conditions, and labor policies influence the psychological age climate and motivation. Additionally, examining private sector organizations and non-profit entities can uncover unique organizational dynamics and practices that support or hinder older workers’ engagement and productivity.

One promising approach is to engage in interdisciplinary and multinational network-building projects within the realms of work and organizational psychology and human resource management (WOP/HRM). Such initiatives, like the current efforts focused on the aging workforce, aim to foster scientific excellence and facilitate the translation of research into practical and societal impacts (Marcus et al., 2024). By leveraging these collaborative networks, future research can draw on a diverse array of methodologies and perspectives, thus enriching the data and providing deeper insights. This broader scope of research will help validate the findings of the current study and offer practical strategies for fostering a positive age climate in diverse work environments, ultimately enhancing the well-being and productivity of the aging workforce.

Addressing the limitations and expanding the research in the suggested directions can further advance this critical area of inquiry, ultimately benefiting both individuals and organizations as they navigate the challenges and opportunities presented by an aging workforce.

### Implications for public policy and organizational management

The implications of these findings for public management and organizational practice are threefold.

First, policymakers and organizational leaders should prioritize the cultivation of inclusive, age-friendly work cultures that recognize and value the contributions of older workers. This may involve implementing anti-age discrimination policies, providing age-inclusive training for managers, and celebrating the achievements of older employees.

The results emphasize the importance of cultivating organizational cultures that value and support older workers. Policymakers should consider implementing regulations, guidelines, or incentive programs that encourage employers to foster more positive psychological age climates. Governments can play a role in developing and disseminating tools, training programs, and best practices to help organizations create work environments that meet the needs of their aging workforce. This could include guidelines on implementing flexible work arrangements, mentorship programs, and age-inclusive management practices (Wissemann et al., 2022).

Policymakers should strengthen legal protections against age discrimination and ensure effective enforcement mechanisms. This can help create a societal climate that recognizes the value of older workers and discourages discriminatory practices.

Policies that promote continuous skills development and training opportunities for workers of all ages can enhance the adaptability and employability of older individuals, supporting their motivation to remain in the workforce.

Second, public and organizational initiatives should focus on enhancing the autonomy and social support available to aged workers. This could include measures such as offering flexible work arrangements, mentorship programs, and opportunities for knowledge sharing and social interaction (Pak et al., 2023). By empowering older workers and fostering a sense of belonging, organizations can better harness the potential of their aging workforce.

The findings suggest that providing aged workers with greater job autonomy and social resources within the workplace can be particularly effective in enhancing their motivation to continue working. Organizations should consider implementing initiatives such as flexible work arrangements, job crafting opportunities, and peer mentorship programs.

Employers should actively work to create organizational cultures that celebrate the contributions of older workers and actively combat age-related biases. This may involve implementing comprehensive anti-discrimination policies that specifically address ageism and promote equality. Providing age-inclusive management training can help managers understand the unique strengths and needs of older workers, fostering a more supportive and inclusive environment. Recognizing the achievements of older employees through awards, public acknowledgments, and career advancement opportunities can significantly enhance their sense of value and motivation (Innocenti et al., 2023).

In addition to these measures, organizations can promote intergenerational collaboration by creating mentorship programs where experienced older workers can share their knowledge with younger colleagues. This not only leverages the expertise of older employees but also fosters mutual respect and understanding across different age groups. Offering flexible work arrangements, such as part-time schedules or telecommuting options, can also accommodate the varying needs of older workers, helping them maintain a healthy work-life balance. Providing opportunities for continuous learning and professional development can further support older employees in staying current with industry trends and enhancing their skills, thereby boosting their confidence and engagement. Regularly soliciting feedback from older workers about their work environment and acting on their suggestions can also improve the psychological age climate, ensuring that the workplace evolves in a way that meets their needs and preferences. By adopting these strategies, employers can create a more inclusive, respectful, and motivating workplace for older employees.

While the study did not find significant mediating effects for skill-tailored work design and ergonomic conditions, these factors remain important considerations for supporting an aging workforce. Organizations should collaborate with older workers to identify and address any physical or cognitive challenges they may face, adapting job requirements and work environments accordingly (Blank, 2024).

The findings suggest that a combination of organizational climate, job-related resources, and physical work environment factors can contribute to the motivation of older workers. Employers should therefore take a comprehensive approach, addressing multiple dimensions to create a supportive ecosystem for their aging workforce (Nygaard et al., 2022).

By aligning public policy and organizational practices with the needs and preferences of older workers, policymakers and employers can more effectively retain and engage this valuable segment of the workforce, ultimately benefiting both individuals and society as a whole.

Moreover, while job design and physical work environments are important considerations, the findings suggest that these factors may not be the most critical drivers of motivation to continue working among older employees. Policymakers and organizations should consider a more holistic approach that balances job-related accommodations with the cultivation of a positive, age-inclusive organizational climate.

Organizations can implement several strategies to effectively offer flexible work arrangements for their older workers. Organizations should engage with older workers to understand their specific needs, preferences, and constraints around work arrangements. This includes considering factors such as caregiving responsibilities, health concerns, and desired work-life balance. By tailoring flexible options based on the unique requirements of the older workforce, organizations can better meet the needs of this demographic.

Developing clear and transparent policies that outline the various flexible work options available, such as part-time work, job-sharing, phased retirement, and remote/hybrid work, is crucial. Ensuring that these policies are communicated effectively and consistently across the organization, and training managers on how to effectively manage and support older workers with flexible arrangements, can help facilitate the implementation of these programs (Liou, 2024).

Offering older workers, the ability to adjust their start and end times, as well as the option to work compressed workweeks or have flexible schedules, can provide the necessary accommodations. Providing the necessary technological and logistical support to enable remote or hybrid work arrangements, while ensuring that flexible scheduling does not disadvantage older workers in terms of career advancement or access to training and development opportunities, is also important.

Developing programs that allow older workers to gradually reduce their workload and transition into retirement, rather than abruptly leaving the workforce, can be beneficial. Offering a range of options, such as reduced work hours, job-sharing, or project-based work, to accommodate the varying preferences and needs of older employees, and ensuring that these programs are integrated with the organization’s broader succession planning and knowledge transfer strategies, can help create a smooth transition (Fasbender et al., 2022).

Regularly soliciting feedback from older workers to assess the effectiveness and satisfaction with the flexible work arrangements, continuously refining and improving the policies and programs based on employee input and changing organizational needs, offering training and resources to help older workers adapt to and thrive in flexible work arrangements, and encouraging open communication and collaboration between older workers, their managers, and HR to address any challenges or concerns, can all contribute to the success of these initiatives.

By adopting a comprehensive and tailored approach to flexible work arrangements, organizations can better support and retain their older workers, ultimately benefiting from their valuable skills, experience, and institutional knowledge.

## Conclusion

This study elucidates the influence of psychological age climate on the motivation of aged workers in China, demonstrating that a positive age climate significantly enhances their motivation to remain employed. The results substantiate the hypothesis that developing an inclusive organizational culture is pivotal for retaining aged workers, a necessity in the context of a globally aging population.

The research identifies autonomy arrangements and social support as critical mediators that facilitate the translation of a positive psychological age climate into increased work motivation. This indicates that providing aged workers with greater job autonomy and robust social resources within the workplace is crucial for leveraging the benefits of a favorable age climate.

Contrary to some existing research, this study did not find support for the mediating roles of job design adapted to aged workers’ skills and ergonomic working conditions. This divergence suggests that in the specific cultural and organizational context of China, where collective values and respect for elder wisdom are prominent, the direct provision of autonomy and social support may exert a more immediate impact on aged workers’ motivation than job design and ergonomic factors.

These findings imply that public policymakers and organizational leaders should devise strategies that focus on fostering positive age climates and enhancing social and autonomy supports as effective means to retain and engage older workers. Tailoring these strategies to accommodate the unique cultural and organizational contexts can optimize their effectiveness.

In conclusion, the study advocates for a comprehensive approach in public management and organizational policies that address the specific needs of the aging workforce. Such an approach is essential for maximizing both individual and organizational outcomes in an era of demographic aging.

## Data availability statement

The raw data supporting the conclusions of this article will be made available by the authors, without undue reservation.

## Ethics statement

The studies involving humans were approved by Ethical Committee of the Inner Mongolia University. The studies were conducted in accordance with the local legislation and institutional requirements. The participants provided their written informed consent to participate in this study.

## Author contributions

ZW: Writing – review & editing, Writing – original draft, Visualization, Validation, Supervision, Software, Resources, Project administration, Methodology, Investigation, Funding acquisition, Formal analysis, Data curation, Conceptualization. JF: Writing – review & editing, Writing – original draft, Visualization, Validation, Supervision, Software, Resources, Project administration, Methodology, Investigation, Funding acquisition, Formal analysis, Data curation, Conceptualization. WB: Writing – review & editing, Writing – original draft, Visualization, Validation, Supervision, Software, Resources, Project administration, Methodology, Investigation, Funding acquisition, Formal analysis, Data curation, Conceptualization.

## Appendix

**Table A1:** Full list of items of the instruments used in the survey.

Scale	Examples of items
1. Psychological age climate	In my company, older employees are seen as... Cooperative Reliable Resilient Open Minded Creative Risk-Loving Competent Flexible Motivated Effective Goal Oriented
2. Autonomy	The job allows me to make my own decisions about how to organize it. The job gives me the opportunity to use my initiative or judgment in performing it. The job allows me to make decisions about the methods I use to carry it out.
3. Skill adaptation	The job requires a variety of skills. The job requires the use of different skills to perform it. The job requires the utilization of a variety of complex or high-level skills. The job requires the use of diverse skills.
4. Social support	In my job, I have the opportunity to develop good friendships. In my job, I have the opportunity to meet other people. In my job, I have the opportunity to interact with others. My supervisor is concerned about the well-being of the people who work for him or her. The people I work with care about me personally. The people I work with are friendly.
5. Ergonomic conditions	The seating arrangement at work is adequate (e.g., ample opportunities to sit, comfortable chairs, good postural support). The workplace accommodates personal differences in terms of space, reach, eye height, legroom, etc. The job involves excessive effort to reach things.
6. Continued working intention	I expect to continue working as long as possible after my official retirement age. Barring unforeseen circumstances, I would remain working as long as possible
